# Retraction Note: Low prices and high regret: how pricing influences regret at all-you-can-eat buffets

**DOI:** 10.1186/s40795-017-0195-6

**Published:** 2017-09-15

**Authors:** Özge Siğirci, Brian Wansink

**Affiliations:** 1 Marmara University, Institute of Social Sciences, Istanbul, Turkey; 2 Charles H. Dyson School of Applied Economics and Management, Cornell University, 112 Warren Hall, Ithaca, NY 14853 USA

## Retraction

The Editor is retracting this article [1] because concerns have been raised [2] after publication with respect to the analysis of the data reported. The authors have been offered the opportunity to submit a new manuscript for peer review. The authors do not agree with this retraction.
